# Terrestrial locomotion energy costs vary considerably between species: no evidence that this is explained by rate of leg force production or ecology

**DOI:** 10.1038/s41598-018-36565-z

**Published:** 2019-01-24

**Authors:** Lewis G. Halsey, Craig R. White

**Affiliations:** 1 Centre for Research in Ecology, Evolution and Behaviour, Department of Life Sciences, University of Roehampton, Holybourne Avenue, London, SW15 4JD UK; 2 Centre for Geometric Biology, School of Biological Sciences, Monash University, Melbourne, Victoria 3800 Australia

## Abstract

Inter-specifically, relative energy costs of terrestrial transport vary several-fold. Many pair-wise differences of locomotor costs between similarly-sized species are considerable, and are yet to be explained by morphology or gait kinematics. Foot contact time, a proxy for rate of force production, is a strong predictor of locomotor energy costs across species of different size and might predict variability between similarly sized species. We tested for a relationship between foot contact time and metabolic rate during locomotion from published data. We investigated the phylogenetic correlation between energy expenditure rate and foot contact time, conditioned on fixed effects of mass and speed. Foot contact time does not explain variance in rate of energy expenditure during locomotion, once speed and body size are accounted for. Thus, perhaps surprisingly, inter-specific differences in the mass-independent net cost of terrestrial transport (NCOT) are not explained by rates of force production. We also tested for relationships between locomotor energy costs and eco-physiological variables. NCOT did not relate to any of the tested eco-physiological variables; we thus conclude either that interspecific differences in transport cost have no influence on macroecological and macrophysiological patterns, or that NCOT is a poor indicator of animal energy expenditure beyond the treadmill.

## Introduction

When moving around, larger animals enjoy economies of scale over smaller animals; per unit mass they expend less energy to travel a given distance^1–5^. Yet terrestrial animals across a great range of sizes have the same mass-independent mechanical cost of transport^6^. Thus when running, the muscles of smaller animals must be employed less efficiently than those of larger animals^7^. Since at least the 1980s^2^ there has been a drive to explain why. Most notably, Taylor *et al*.^8^ and others sought to explain their observation that during terrestrial locomotion the energy cost to apply a unit of force to the ground is higher in smaller animals. When terrestrial animals are on the move their primary energy cost comes from the need to support their body weight^9,10^, which is achieved through muscular contractions generating the vertical component of the ground reaction force each time a limb makes contact with the substrate. In absolute terms smaller animals have shorter legs (Kram and Taylor 1990) and therefore higher stride frequencies than do larger animals^11–14^, and in turn the duration that their feet are in contact with the ground per step is lower. Thus smaller animals must apply the necessary ground reaction forces more quickly, which is believed to require the employment of muscle fibres that are less energetically efficient^9,15,16^.

While the relationship between the energy costs of transport and body mass is strong (e.g. r^2^ = 0.85^17^) at any given mass there is a several-fold range in metabolic costs per unit distance (net cost of transport; NCOT) between species while walking on the laboratory treadmill^1,4,18,19^. For example, both the wild turkey and the marabou stork weigh around 4 kg, yet the former consumes 0.41 mlO_2_ kg^−1^ per m^20^ while the stork consumes around eight times that amount^21^. Given that locomotion costs can represent a substantial proportion of an animal’s daily energy budget^22–27^, these large differences in energy expenditure between species are surprising. From the perspective of optimal foraging^28,29^ and energy allocation theories^30,31^, such vast variability seems counter to the concept that animals have evolved to economise their daily energy expenditure, within the constraints of their ecology, in order to maximise the energy they have remaining, which can be directed towards reproduction^32^. In turn, this variability calls into question the importance of energetics, or at least locomotory energy expenditure, in shaping an animal’s ecology^33^. Yet there has been little research into the ecological implications of these differences in locomotion cost exhibited by similarly sized species.

It has been known for a long time that penguins have a high cost of transport^34,35^. Initially, this was believed explicable by their waddling gait. However, Griffin and Kram^36^ posited that penguins expend a lot of energy when walking because of their relatively short hind limbs resulting in low foot contact times, and thus high rates of force production^9^. They therefore argued that the proposed explanation for the greater relative NCOT in smaller animals might also explain the greater relative NCOT in the short-legged penguins^4^. By extension, the inference has been made that in general, species exhibiting a high NCOT for their size might have relatively short legs and, conversely, animals that display particularly good energy economies tend to have long legs (see also^11^). Kram and Taylor’s (1990) function relating metabolic rate of locomotion to foot contact time has successfully predicted gait-metabolic cost relationships in various contexts^37–39^. However, there has yet to be a substantial across-species analysis to test the hypothesis of a relationship between foot contact time and the cost of transport. Thus although it is often assumed to be the case, we do not know whether rate of force production describes variation in the cost of transport inter-specifically.

Limb morphology was included as an explicit determinant of locomotion energy costs in a biomechanical model (LiMb) developed by Pontzer^40^. The LiMb model recognises that energy expenditure during terrestrial locomotion, while mostly explained by the costs of generating the vertical component of the ground reaction force, also includes the costs of the horizontal component associated with cyclical braking and propulsion^41^ and the costs to swing the limbs^42,43^. The model was initially applied to an intra-specific analysis of human participants and performed better at predicting running metabolic rate than did foot contact time^40^. A similar conclusion arose from intra-specific analyses of two quadruped species: dogs and goats^44^.

The LiMb model requires the input of three parameters, two of which are the energy cost of swinging the limb and the excursion angle of the limb during the stance phase. Both of these are generally only obtainable under laboratory conditions. However, the model predicts that over a wide range of body sizes the other and most easily measured input parameter, effective limb length (the functional length of the limb as a mechanical strut), will accurately estimate locomotion costs because it is the only one of the three parameters that scales with body mass. Evidence supporting this prediction came from an across-species study; log-transformed effective limb length explained 98% of the variance in log-transformed mass-specific locomotor costs (as opposed to 94% explained by log-transformed body mass)^45^, in turn supporting aforementioned previous work suggesting that the magnitude and frequency of muscle forces generated to counter gravitational acceleration is a key determinant of NCOT across species^36–39^ (cf^6^).

**Figure 1:**
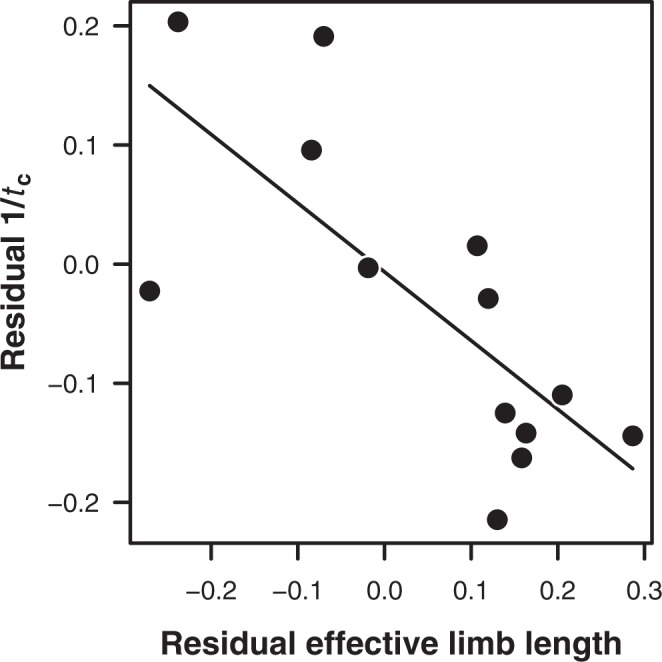
An inter-specific regression of the residuals of log(effective limb length) on log(mass) against the residuals of log(1/contact time [*t*_c_]) on log(mass) and log(speed) from the dataset of the present study returned R^2^ = 0.61 (r = −0.78, N = 13). Data for effective limb length are from Pontzer^45^ and Halsey^72^; data for contact time are provided online (Supplementary).

However, despite this impressively high coefficient of variation, similarly to the aforementioned relationships between cost of transport and body mass, the use of log-transformed data across a wide mass range masks the absolute size of the residuals; a third of the residuals have a magnitude greater than 20%. Moreover, many comparisons between relevant pairs of species within the dataset do not support the premise that longer effective limbs associate with lower transport costs^45^. This might be explained by effective limb length not relating to rate of force production, for example because it does not account for the excursion angle of the limb (which as mentioned previously is an additional variable in the LiMb model); excursion angle dominates the variation in locomotor cost at the intraspecific level^40,45^. This proposed explanation is indirectly evidenced by an across-species regression, based on the present dataset, of relative effective limb length against rate of force production represented by relative foot contact time; accounting for speed, this regression returned a comparatively low R^2^ of 0.61 (Fig. 1).

Foot contact time is mathematically more closely associated with rate of force production against the ground than is effective limb length. Assuming that rate of force production is a key driver of NCOT across species then foot contact time is perhaps the proxy likely to associate best with variations in NCOT between similarly-sized species despite the great variation in morphologies and walking gaits they exhibit. At the least, it seems reasonable to re-asses this hypothesis, drawing upon the more extensive data sets for foot contact times and metabolic rates of terrestrial animals on the move that are now available. We collated these data to investigate whether, across species, rate of force production represented by foot contact time is predictive of a species’ locomotion costs. Using a phylogenetically informed approach, we focussed on birds and mammals (since data for these taxa are by far the most prevalent) to test for an interspecific relationship between metabolic rate during locomotion and foot contact time, while also recognising and accounting for the relationships between each of these variables and both body mass and locomotion speed, using phylogenetic mixed models to formally incorporate the non-independence associated with multiple measurements of a single species. We then expanded the analysis to consider additional predictors of interspecific variation in NCOT, testing for correlations between NCOT and a host of putative eco-physiological correlates, to examine the ecological relevance of NCOT beyond the laboratory. For this analysis we focussed on mammals to take advantage of the rich data sets published by Jones *et al*.^46^ and multiple other sources^24,47–52^.

## Materials and Methods

A total of 288 observations of mass-specific rate of oxygen consumption (${\dot{V}}_{{O}_{2}}$, ml kg^−1^ min^−1^) and 126 observations of foot contact time (*t*_c_, s) during pedestrian locomotion were obtained for 21 species (combining data for large leghorn and bantam leghorn chickens, and for musculus and musculus longshanks mice; Table 1) mainly from published studies^9,15,34,36,53–71^. The species in this study represent a mass range of 32 g to 467 kg. Previously unpublished kinematic data for king penguins are included as part of the present study. In most cases collation of data from the literature required data extraction from digitised versions of published figures. We used data for ${\dot{V}}_{{O}_{2}}$ (i.e. rather than NCOT) to test for the relationship between transport costs and 1/*t*_c_ because the relationship between speed and 1/*t*_c_ is not always linear, and it is therefore not possible to calculate species-specific speed-independent values for foot contact times. Because the y-intercept of the relationship between ${\dot{V}}_{{O}_{2}}$ and speed is greater than zero and typically also greater than resting metabolic rates e.g.^72^, for each data set we calculated a linear regression relating ${\dot{V}}_{{O}_{2}}$ and speed, and subtracted the y-intercept of this relationship from each value of ${\dot{V}}_{{O}_{2}}$ in the data set, following previous studies (e.g^15^.). Our data for ${\dot{V}}_{{O}_{2}}$ therefore represent only the incremental increases in ${\dot{V}}_{{O}_{2}}$ associated with increases in speed.

**Table 1:** Summary of species and variables collated to test the hypothesis that, for a given body mass, an animal’s NCOT is explained by its foot contact time.

Common name	Latin name	Speed range (m s^−1^)	V’O_2_ range (ml kg^−1^ min^−1^)	Foot contact time range (s)	Mass range (kg)	y intercept range (ml kg^−1^ min^−1^)	References
Bobwhite quail	Colinus virginianus	0.28–0.69	38.55–62.00	0.24–0.33	0.13–0.19	21.97–62.00	Fedak *et al*.^53^ Hoyt^54^
Kangaroo rat	Dipodomys merriami	1.03–2.85	9.91–118.25	0.04–0.06	0.032	−47.63	Thomson *et al*.^55^ Kram and Taylo^9^
Squirrel	Spermophilus tridecemlineatus	0.60–2.62	101.84–161.02	0.05–0.14	0.21–0.23	94.35	Hoyt and Kenagy^56^ Hoyt^54^
Spring hare	Pedetes capensis	0.57–2.83	41.25–100.38	0.12–0.15	3	30.72	Seeherman *et al*.^57^ Hoyt^54^
Guinea fowl	Numida meleagris	0.41–1.99	33.24–70.78	0.20–0.33	1.3–1.44	23.14	Fedak *et al*.^53^ Hoyt^54^
Dog	Canis familiaris	1.43–7.04	19.02–57.07	0.08–0.24	24–25.8	6.20	Cerretelli *et al*.^58^ Hoyt^54^
Turkey	Meleagris gallopavo	0.69–3.50	26.84–77.41	0.17–0.33	4.31–5.3	10.68	Fedak *et al*.^53^ Hoyt^54^
Horse/pony	Equus caballus	2.00–7.01	15.62–43.54	0.11–0.39	140–467	0.664	Wickler *et al*.^60^ Hoyt and Taylor^59^ Hoyt^54^
Rhea	Rhea Americana	0.49–3.80	16.23–76.95	0.21–0.55	19.9–22	5.83	Fedak *et al*.^53^ Hoyt^54^
Emu	Dromaius novaehollandiae	1.50–4.00	20.79–37.67	0.25–0.62	40.1	4.74	Roberts *et al*.^15^ Watson *et al*.^61^ Hoyt^54^
Human	Homo sapiens	2.19–4.02	30.09–46.41	0.25–0.38	78.88–80.20	−0.51	Bransford and Howley^62^ Hoyt^54^
Rat-kangaroo	Bettongia penicillata	1.10–6.20	81.80–114.39	0.05–0.10	0.97	70.95	Webster and Dawson^63^
Peacock	Pavo cristatus	0.5–1.01	9.15–16.61	0.49–0.79	4.58	1.43	Wilkinson *et al*.^64^
King penguin	Aptenodytes patagonicus	0.08–0.50	15.91–25.63	0.56–0.79	11.5–11.65	13.25	Fahlman *et al*.^65^ Present study
Svalbard rock ptarmigan	Lagopus muta hyperborean	0.22–0.75	33.76–46.22	0.30–0.55	0.73	29.3	Lees *et al*.^66^
Barnacle geese	Branta leucopsis	0.24–1.25	28.52–64.81	0.21–0.81	1.79	20.46	Nudds and Codd^67^
Great cormorant	Phalacrocorax carbo	0.08–0.5	29.95–49.88	0.46–1.26	2.26	29.21	White *et al*.^68^
Platypus	Ornithorhynchus anatinus	0.19–1.08	11.98–29.05	0.18–1.30	1.40	9.59	Fish *et al*.^70^
Emperor penguin	Aptenodytes forsteri	0.28–2.72	10.81–30.25	0.33–0.54	20.79–21	7.26	Griffin and Kram^36^ Pinshow *et al*.^34^
Large leghorn/ bantam leghorn	Gallus gallus	0.28–0.69	29.72–52.20	0.36–0.84	1.39–1.92	14.11	Rose *et al*.^69^
Laboratory mouse	Mus musculus (longshanks)	0.08–0.33	82.61–93.86	0.11–0.18	0.04	79.00–79.98	Sparrow^71^

Data for NCOT were obtained from a published compilation^4^. Data for putative eco-physiological correlates of NCOT were obtained from the PanTheria database^46^, namely: home range size, geographical range, group size, terrestriality, diet breadth, trophic level and habitat breadth. Additional variables were obtained from other sources: basal metabolic rate^47^, field metabolic rate^48^, maximum aerobic metabolic rate^49^, daily movement distance^24^, maximum running speed^50^, and body fat^51,52^. Absolute aerobic scope was calculated as the difference between maximum aerobic metabolic rate and basal metabolic rate. Factorial aerobic scope was calculated as the ratio of maximum aerobic metabolic rate to basal metabolic rate. Activity metabolic rate was calculated as the difference between field metabolic rate and basal metabolic rate^73,74^.

The speed- and size-independent relationship between log_10_-transformed mass-specific rate of oxygen consumption (${\dot{V}}_{{O}_{2}}$, ml kg^−1^ min^−1^) and log_10_-transformed 1/*t*_c_ (s^−1^) was analysed using multivariate phylogenetic mixed models^75–77^. Phylogenetic mixed models were implemented in the ASReml-R v3.0^78^ package of R v3.0.2^79^, with inverse relatedness matrices calculated from phylogenetic covariance matrices using the MCMCglmm package v2.21^80^. Phylogenetic mixed models were selected over the more commonly used methods of independent contrasts^81,82^ and phylogenetic generalised least squares^82,83^ because the former can formally incorporate phylogenetic non-independence as well as non-independence associated with multiple measurements of single species (i.e. measurements of ${\dot{V}}_{{O}_{2}}$ and *t*_c_ for a single species running at multiple speeds). Phylogenetic mixed models are an analogue of the mixed model from quantitative genetics, which partitions phenotypes of related individuals into heritable (additive genetic) and non-heritable components to estimate inter-specific variances and covariances between traits^77^.

We used multivariate phylogenetic mixed models to examine the phylogenetic correlation among the traits ${\dot{V}}_{{O}_{2}}$ and 1/*t*_c_, accounting for either body mass or speed, or accounting for both body mass and speed (a phylogenetic correlation between two traits represents the proportion of variance that these two traits share due to phylogenetic relatedness, after accounting for any fixed effects in the model). The multivariate models included log_10_(${\dot{V}}_{{O}_{2}}$) and log_10_(1/*t*_c_) as response variables, either or both of log_10_-transformed speed (*U*, m s^−1^) and log_10_-transformed body mass (*M*, kg) as fixed effects, and phylogenetic relatedness as a random effect (it was not possible to include species identity as a random effect in the multivariate models because there was insufficient data to fit covariance matrices for both phylogenetic relatedness and species identity). Completely parameterised (unstructured) (co)variance matrices were specified for the random effect associated with the phylogeny, as well as the residuals. The significance of phylogenetic correlations between log_10_(${\dot{V}}_{{O}_{2}}$) and log_10_(1/*t*_c_), conditioned on the fixed effects, were inferred by determining if the phylogenetic covariance between log_10_(${\dot{V}}_{{O}_{2}}$) and log_10_(1/*t*_c_) differed significantly from zero by using a likelihood ratio test to compare models with and without the appropriate covariances fixed at zero. Approximate standard errors for phylogenetic correlations were calculated using the R ‘pin’ function^84^.

**Figure 2:**
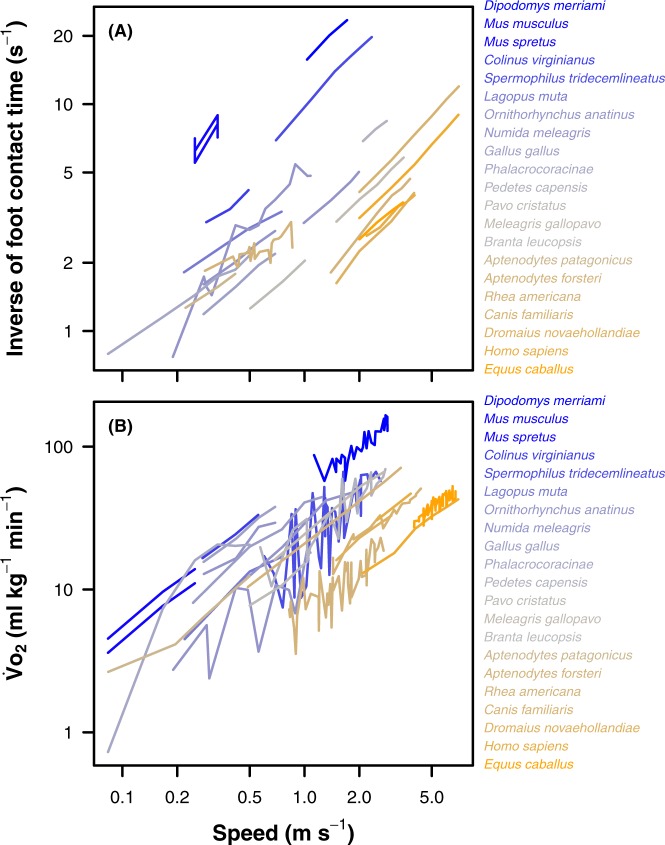
Relationships between (**A**) inverse of foot contact time and (**B**) rate of oxygen consumption, against locomotion speed. Each line in each panel represents an individual data set for a distinct species (N = 21), which are coloured by body mass from small (blue) to large (orange). Note that data are shown on log-transformed axes. The data set includes a total of 414 observations, and all data are provided in the online information.

Before running the multivariate phylogenetic mixed model, we first visually verified that the relationships between log_10_(*U*) and both of log_10_(${\dot{V}}_{{O}_{2}}$) and log_10_(1/*t*_c_) were approximately linear for all species (Fig. 2), and used univariate phylogenetic mixed models to test for significant interactions between the fixed effects of log_10_(*U*) and log_10_(*M*) for both log_10_(${\dot{V}}_{{O}_{2}}$) and log_10_(1/*t*_c_). The significance of fixed effects was tested using Wald-type *F*-tests with conditional sums of squares and denominator degrees of freedom calculated according to Kenward and Roger^85^. Phylogenetic heritability (*h*^2^), a measure of phylogenetic correlation equivalent to Pagel’s^86^ λ^77^, was estimated as the proportion of variance attributable to the random effect of phylogeny; the proportion of variance attributable to species identity independent of phylogeny was also calculated. The significance of phylogenetic heritability and the random effect of species identity were assessed using likelihood ratio tests to compare models with and without the random effects. Approximate standard errors for the estimate of phylogenetic heritability were calculated using the R ‘pin’ function^84^.

To test for correlations between NCOT and each of the putative eco-physiological correlates of NCOT, because there was only a single value per species we used the phylogenetic generalised least squares (PGLS) method^82,83^ and the ‘ape’ v3.2^87^ and ‘caper’ v0.5.2^88^ packages of R v3.0.2^79^, with Pagel’s λ^86^ estimated using maximum likelihood. Body mass was included as a covariate in all analyses, and all data were log_10_-transformed for analysis. The PGLS method is equivalent to univariate phylogenetic mixed models when only one response value per species is analysed, as is the case for NCOT data.

The phylogeny used for all analyses was obtained from the open tree of life^89^ using R v3.2.2 and the R package ‘rotl’^90^, with branch lengths estimated using the arbitrary method of Grafen^83^ in ‘ape’ v3.2^87^.

Parameter and variance component estimates are shown ± SE, unless otherwise noted, and α was set at 0.05 for all tests. Multiple comparison corrections were not performed^91^.

## Results

Assessed using univariate phylogenetic mixed models, there was no interaction between log_10_(*M*) and log_10_(*U*) as predictors of log_10_(${\dot{V}}_{{O}_{2}}$) (Table 2) or log_10_(1/*t*_c_) (Table 3) during treadmill locomotion, indicating that there is no need to include an interaction terms in the multivariate phylogenetic mixed model. With only the additive effects of log_10_(*M*) and log_10_(*U*) included, there was no significant phylogenetic heritability for log_10_(${\dot{V}}_{{O}_{2}}$). However, there was significant among-species variation unrelated to phylogeny (Table 4), indicating that there are consistent mass- and speed-independent differences among-species in log_10_(${\dot{V}}_{{O}_{2}}$) that are not related to patterns of phylogenetic relatedness (i.e. related species are not more similar than unrelated species). Conversely, for log_10_(1/*t*_c_) there was significant phylogenetic heritability but no significant among-species variation unrelated to phylogeny (Table 5); this indicates that there are significant speed- and mass-independent differences in log_10_(1/*t*_c_) among species, but that related species are more similar than unrelated ones.

**Table 2:** Parameter estimates for univariate phylogenetic mixed models describing the effect of speed (*U*, m s^−1^), body mass (*M*, kg), and their interaction on rate of oxygen consumption (${\dot{V}}_{{O}_{2}}$, ml kg^−1^ min^−1^).

Term	Estimate	SE	*F* (df)	P
Intercept	1.416	0.063	510 (1,1.6)	0.005
log_10_(*M*)	−0.178	0.037	23.0 (1,25.1)	<0.001
log_10_(*U*)	1.027	0.044	628 (1,273.1)	<0.001
log_10_(*M*) ∗ log_10_(*U*)	0.022	0.036	0.35 (1,173.9)	0.55
*Phylogeny*	*0*.*0063*	*0*.*0127*		
*Species*	*0*.*0273*	*0*.*0116*		
*Residual*	*0*.*0140*	*0*.*0012*		

Fixed = log_10_(${\dot{V}}_{{O}_{2}}$) ~ log_10_(*M*) + log_10_(*U*) + log_10_(*M*) ∗ log_10_(*U*). The model includes random effects of phylogeny and species, which are used to determine the variance associated with the phylogeny and the variance associated with species-specific differences not associated with phylogeny, respectively, conditioned on the fixed effects. *h*^2^ is the proportion of variance, conditioned on the fixed effects, accounted for by the random effect of phylogeny (shown ± SE), and is equivalent to Pagel’s λ^77,86^; *χ*^2^ is the test statistic used to determine if the variance estimates for the random effects of phylogeny and species are significantly greater than zero. Estimates associated with ‘Phylogeny’ and ‘Species’ are estimates of the variances associated with each of these random effects, conditioned on the fixed effects, and the estimate associated with ‘Residual’ is the residual variance.

Phylogenetic *h*^2^ = 0.13 ± 0.24 (*χ*^2^_1_ = 0.69, P = 0.41). Proportion of variance accounted for by ‘*Species*’: 0.57 ± 0.21 (*χ*^2^_1_ = 14.7, P < 0.001).

**Table 3:** Parameter estimates for univariate phylogenetic mixed models describing the effect of speed (*U*, m s^−1^), body mass (*M*, kg), and their interaction on the inverse of foot contact time (*t*_c_, s). Fixed = log_10_(1/*t*_c_) ~ log_10_(*M*) + log_10_(*U*) + log_10_(*M*) ∗ log_10_(*U*). For further model details, see the title for Table 2.

Term	Estimate	SE	*F* (df)	P
Intercept	0.719	0.010	29.6 (1,8.0)	<0.001
log_10_(*M*)	−0.284	0.021	26.3 (1,27.7)	<0.001
log_10_(*U*)	0.753	0.029	874 (1,120)	<0.001
log_10_(*M*) ∗ log_10_(*U*)	0.002	0.029	0.0036 (1,100)	0.95
*Phylogeny*	*0*.*0295*	*0*.*0152*		
*Species*	*0*.*0007*	*0*.*0019*		
*Residual*	*0*.*0025*	*0*.*0004*		

Phylogenetic *h*^2^ = 0.90 ± 0.09 (*χ*^2^_1_ = 12.6, P < 0.001). Proportion of variance accounted for by ‘*Species*’: 0.02 ± 0.06 (*χ*^2^_1_ = 0.160, P = 0.69).

**Table 4:** Table 2. Parameter estimates for univariate phylogenetic mixed models describing the effect of speed (*U*, m s^−1^) and body mass (*M*, kg) on rate of oxygen consumption (${\dot{V}}_{{O}_{2}}$, ml kg^−1^ min^−1^). Fixed = log_10_(${\dot{V}}_{{O}_{2}}$) ~ log_10_(*M*) + log_10_(*U*). For further model details, see the title for Table 2.

Term	Estimate	SE	*F* (df)	P
Intercept	1.421	0.059	572 (1,1.5)	0.006
log_10_(*M*)	−0.173	0.036	23.3 (1,25.2)	<0.001
log_10_(*U*)	1.035	0.041	630 (1,273)	<0.001
*Phylogeny*	*0*.*0053*	*0*.*0119*		
*Species*	*0*.*0276*	*0*.*0116*		
*Residual*	*0*.*0140*	*0*.*0012*		

Phylogenetic *h*^2^ = 0.11 ± 0.24 (*χ*^2^_1_ = 0.50, P = 0.48). Proportion of variance accounted for by ‘*Species*’: 0.59 ± 0.21 (*χ*^2^_1_ = 14.5, P < 0.001).

**Table 5:** Parameter estimates for univariate phylogenetic mixed models describing the effect of speed (*U*, m s^−1^) and body mass (*M*, kg) on the inverse of foot contact time (*t*_c_, s). Fixed = log_10_(1/*t*_c_) ~ log_10_(*M*) + log_10_(*U*). For further model details, see the title for Table 2.

Term	Estimate	SE	*F* (df)	P
Intercept	0.719	0.099	30.0 (1,8.1)	<0.001
log_10_(*M*)	−0.285	0.021	26.5 (1,29.2)	<0.001
log_10_(*U*)	0.754	0.025	880 (1,122)	<0.001
*Phylogeny*	*0*.*0291*	*0*.*0150*		
*Species*	*0*.*0007*	*0*.*0019*		
*Residual*	*0*.*0025*	*0*.*0004*		

Phylogenetic *h*^2^ = 0.90 ± 0.09 (*χ*^2^_1_ = 13.2, P < 0.001). Proportion of variance accounted for by ‘*Species*’: 0.02 ± 0.06 (*χ*^2^_1_ = 0.184, P = 0.67).

**Figure 3:**
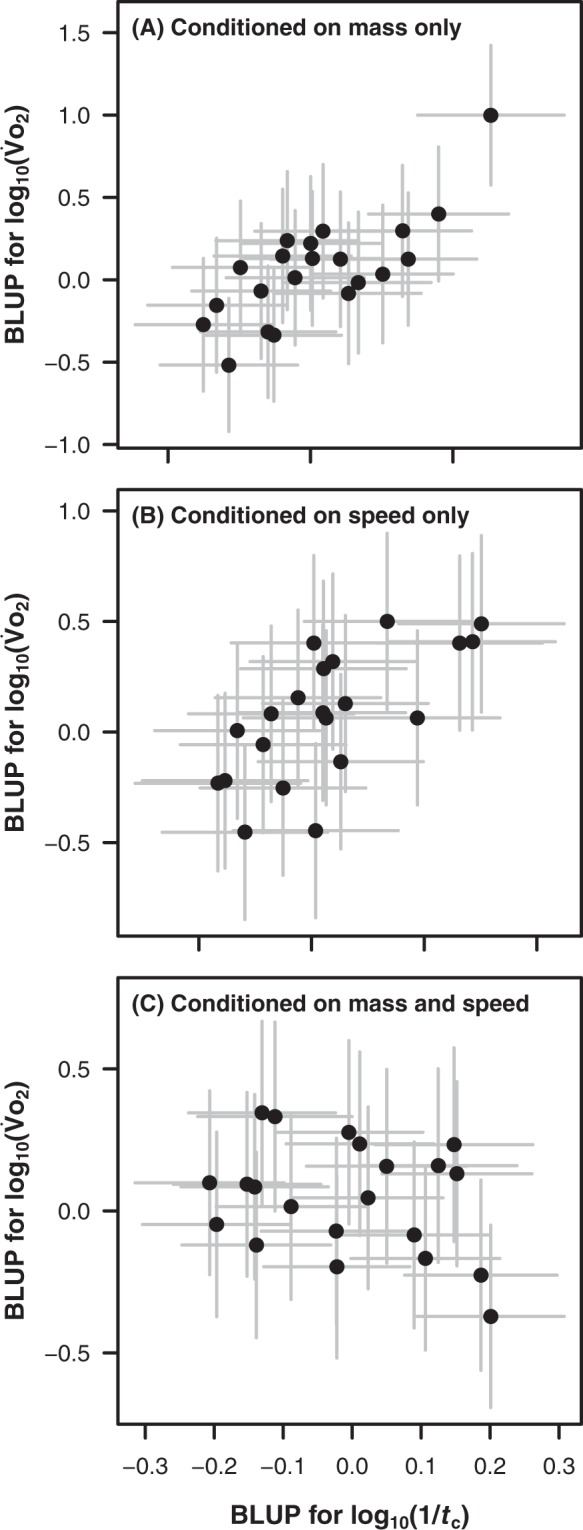
Relationships between log_10_(${\dot{V}}_{{O}_{2}}$) and log(1/contact time [*t*_c_]), accounting for (**A**) log_10_(mass), (**B**) log_10_(speed), and (**C**) both log_10_(mass) and log_10_(speed). ${\dot{V}}_{{O}_{2}}$ has units of ml kg^−1^ min^−1^ and tc has units of s. Each point is the best linear unbiased predictor (BLUP) for the random effect of phylogeny for each species (N = 21), shown ±SE, which provides shrunken estimates of the differences between terms and the overall means. The BLUP values are used for visualisation, and quantify the phylogenetic component of each species’ deviation from the overall means; a positive relationship between the BLUPs for log_10_(${\dot{V}}_{{O}_{2}}$) and log_10_(1/*t*_c_) indicates that, once the fixed effects are accounted for, species that evolve a high log_10_(${\dot{V}}_{{O}_{2}}$) also evolve a high log_10_(1/*t*_c_), and vice versa. The choice of x and y axes for these visualisations is arbitrary. The phylogenetic correlations in panels (**A**) and (**B**) are significant (*r* = 0.87 and 0.64, respectively, P ≤ 0.002), the correlation in panel (**C**) is not (*r* = −0.02, P = 0.95).

Assessing multivariate phylogenetic mixed models, the phylogenetic correlation between log_10_(${\dot{V}}_{{O}_{2}}$) and log_10_(1/*t*_c_) was positive and significantly different from zero when accounting for log_10_(*M*) only (*r* = 0.87 ± 0.08, *χ*^2^_1_ = 17.1, P < 0.001, Fig. 3A) and when accounting for log_10_(*U*) only (*r* = 0.64 ± 0.13, *χ*^2^_1_ = 9.64, P = 0.002, Fig. 3B). Thus, on a logarithmic scale, ${\dot{V}}_{{O}_{2}}$ relates to 1/*t*_c_ when accounting for body mass, or when accounting for speed. The correlation was not significantly different from zero when both log_10_(*M*) and log_10_(*U*) were accounted for (*r* = −0.02 ± 0.27, *χ*^2^_1_ = 0.003, P = 0.95, Fig. 3C). Thus ${\dot{V}}_{{O}_{2}}$ no longer relates to 1/*t*_c_ when both body mass and speed are accounted for.

None of the eco-physiological traits were significantly related to NCOT when body mass was accounted for (Table 6).

**Table 6:** Outputs from phylogenetic generalized least squares (PGLS) analysis models to predict log_10_(NCOT), mlO_2_ m^−1^

Dependent variable, units	N	Estimate	SE	t value	P value
Log(Home range), km^2^	51	−0.011	0.023	−0.474	0.638
Log(DMD), km	15	−0.079	0.117	−0.676	0.512
Log(Geographic range), km^2^	68	0.041	0.026	1.615	0.111
Log(Group size), individuals	30	−0.013	0.053	−0.242	0.810
Terrestriality	57	0.032	0.049	0.654	0.516
Diet breadth	63	−0.004	0.011	−0.339	0.736
Trophic level	63	0.021	0.028	0.733	0.466
Habitat breadth	57	−0.005	0.040	−0.115	0.909
Log(BMR), mlO_2_ h^−1^	63	0.010	0.079	0.129	0.898
Log(FMR), mlO_2_ h^−1^	16	−0.012	0.118	−0.105	0.918
Log(MMR), mlO_2_ h^−1^	27	0.032	0.127	0.250	0.805
**Log(AMR), mlO**_**2**_ **h** ^**−1**^					
Log(FAS)	22	−0.002	0.143	−0.014	0.989
Log(AAS)	22	−0.017	0.137	−0.125	0.902
Log(Max. running speed), km h^−1^	19	0.127	0.283	0.450	0.659
Log(fat), g	17	0.090	0.060	1.502	0.155

For all models, body mass was included as a covariate. For all models the maximum likelihood value of λ was 0.

DMD = daily movement distance; BMR = basal metabolic rate; FMR = field metabolic rate; MMR = maximum metabolic rate; AMR = activity metabolic rate; FAS = factorial aerobic scope; AAS = absolute aerobic scope.

## Discussion

Most explanations for animal scaling of mechanical efficiency during locomotion are premised on the concept that energy costs of locomotion derive primarily from the muscular force to support the body and, particularly for larger animals, to accelerate the body as it oscillates through the step cycle^6,40^. Smaller animals have higher stride frequencies due to their shorter limbs, which necessitate higher rates of force generation due to shorter foot contact times^9^. This requires the recruitment of faster, less efficient muscle fibres, which demand higher rates of cross-bridge cycling and Ca^2+^ pumping, and thus use more ATP per gram of active muscle^16,92,93^. Consequently, at a higher stride frequency more energy is expended to deliver the necessary ground reaction force to support the body. Superficially, this theory appears to explain the present results, because foot contact time – a proxy for rate of force production – describes variation in the energy cost of locomotion between species of different sizes locomoting at the same speed (Fig. 3B), as well as of the same size moving at different speeds (Fig. 3A). However, the among-species relationship disappears when both speed and body size are accounted for simultaneously (Fig. 3C). This finding indicates that the apparent relationships among speeds (Fig. 3A) and sizes (Fig. 3B) arise because of the independent effects of speed and size on both foot contact time (Fig. 2A) and ${\dot{V}}_{{O}_{2}}$ (Fig. 2B), rather than because of an among-species link between (speed- and size-independent) foot contact time and ${\dot{V}}_{{O}_{2}}$. Stated another way, for animals of similar size locomoting at similar speeds, there is no among-species relationship between foot contact time and ${\dot{V}}_{{O}_{2}}$. In turn, this suggests, surprisingly, that rate of ground force production is not a substantive influence on NCOT between species. There must therefore be overriding, alternate factors at play determining the often considerable variation in energy cost of transport between species of similar size.

Not only does NCOT not relate inter-specifically to foot contact time, it also does not relate with any of the eco-physiological variables investigated, from home range size to running speed to body fat mass. While such data may be noisy, this consistent lack of correlative evidence raises the question as to whether NCOT – a measure obtained from prescribed and highly controlled protocols – is meaningful beyond the laboratory i.e. meaningful within an ecological context.

In the remainder of the Discussion we first consider what factors other than rate of force generation might explain variation in ${\dot{V}}_{{O}_{2}}$ between species during terrestrial locomotion, and then interpret and consider the implications for the lack of correlation between NCOT and all available eco-physiological variables.

### Possible mechanisms underlying NCOT

The phylogenetically informed estimate of the scaling exponent for the relationship between log_10_(NCOT) and log_10_(body mass) is −0.28^4^. Given that NCOT in that study, as in the current one, is presented in mass-specific terms, the whole-animal scaling exponent for NCOT is 1 −0.28 = 0.72, which is similar to the phylogenetically informed estimate of the scaling exponent of BMR of 0.69 ± 0.01^47^. This similarity in turn raises the question as to whether the scaling laws governing NCOT are the same as those governing BMR. However, similarity of scaling exponents does not mean that two traits are functionally related^94^, and our analyses found no association between NCOT and BMR once the effect of body mass on BMR was accounted for. Thus the relationship between NCOT and mass is not explained by the relationship between BMR and mass.

Morphological differences between species are likely to include the amount of active muscle that is applying force during periods of foot-ground contact^1^ (see also^95^). For example, birds have longer legs than do mammals and thus longer muscle fibres and a greater muscle volume, which can explain the considerably higher energy expenditure of birds for a given rate of force production^15,96^. Effective mechanical advantage based on the ratio of the anatomical moment arm of the muscles to the load arm of the ground force vector^97^ can also vary considerably between species, including when muscle fibre length has been accounted for^98^. Indeed, Pontzer *et al*.^98^ demonstrate that step length, a strong proxy for foot contact time^9^, is only a limited predictor of NCOT across (eight) species of birds and mammals, arguing that this is because of diversity within those species of muscle fibre length and effective mechanical advantage. Similar effective limb lengths in animal species can be represented by diverse arrangements of the limb skeletal structure resulting in differing degrees of upright or crouched stance; the more upright the limb (the more erect the animal’s posture) the lower the cross-sectional area of muscle required to generate sufficient force to counter gravity^37,99^. There may also be differences between species in the relative shortening velocity of active muscle. For example, due to the force-velocity relationship of skeletal muscle, in cases where the fibres are operating at lower shortening velocities a smaller cross-sectional area of muscle would need to be activated to provide the same force as muscles operating at higher shortening velocities^15,100^. Recent work by Cavagna and Legramandi^101^ provides a new approach to considering the biomechanics underpinning the scaling of locomotion efficiencies with size. They demonstrated that there is variability in the hysteresis energy loss experienced by animals during the lower part of the vertical oscillation of the centre of mass. While muscle-tendon units are undergoing their stretch-shortening cycle, in heavier animals a greater role is played by more elastic tendons at the expense of less elastic muscles. Pontzer^102^ also argues for a graded difference across body mass in the mechanics underlying locomotion energy costs, but through a different pathway. He presents a model which indicates that force production becomes a smaller part of the cost, and mechanical work a greater part of the cost, as animals become larger.

In conclusion, while briefer ground-foot contact times may require faster, less energetically efficient muscle fibres, this mechanism appears insufficient to explain speed- and size-independent differences in the energy cost of locomotion between species. Indeed, an interpretation of recent work by Gutmann and Bertram (2017)^103^ is that if species of a similar size have very different stride frequencies at a given locomotion speed, as is apparent from data presented by^54^, then Kram and Taylor’s (1990) relationship between metabolic rate and foot contact time should not hold. Instead, the volume of muscle activated per step^44^, variations in effective mechanical advantage for instance due to posture^102^, muscle contraction efficiency in terms of mechanical power^104^, and/or bigger animals having a lower energy loss by hysteresis during each step^101^, may explain an important proportion of the variation in residual movement costs. Sufficient information across species on duty factor, limb length, musculature and moment arms along with kinematic information will be necessary to investigate the covariance of these factors with the energy cost of locomotion across species. Alternatively, the considerable diversity in the species represented in inter-specific scaling relationships of NCOT perhaps hints that, for any given species, there could be a plethora of reasons explaining its relatively high or low costs of transport and, furthermore, these could be idiosyncratic. Consequently, mechanisms influencing the energy cost of locomotion beyond mass and, for example, effective limb length may not be generalizable.

### Ecological correlates with NCOT

For many terrestrial species, the amount of energy they expend to locomote substantially decreases the energy remaining that they can channel into reproduction^105–107^. Thus animals are expected to optimise the efficiency of their energy expenditure during movement, within certain constraints^108^. In turn, we might predict correlations between species ecology and energy costs of transport. Yet our analysis on mammals found no relationships between NCOT and a host of ecological variables once body mass, which accounts for the fact that bigger animals have a lower NCOT and can range further^102^, was accounted for. Effectively, our analyses show that for different species of the same size, home range size, daily movement distance, geographic range, group size, terrestriality, diet breadth, trophic level and habitat breadth do not relate to NCOT. Furthermore, despite the fact that animals which can run faster or more energetically cheaply perhaps have the opportunity to roam more extensively and those with greater energy output might be predicted to carry greater energy stores to buffer against short falls^109^, neither maximum running speed nor percentage body fat levels were related to NCOT. Finally, NCOT was also not related to FMR, MMR, AMR or aerobic scope.

Thus NCOT appears to be disconnected from animal foraging behaviour, broader measures of energetics and varying aspects of ecology. This either calls into question the importance of locomotion energy expenditure in influencing an animal’s ecology, or the relevance of NCOT for understanding the locomotion energetics of terrestrial animals beyond the realms of biomechanics or comparisons among many animals of varying size. If either or both possibilities are true, in turn it is important to understand why NCOT has little ecological relevance. Pontzer^110^ argues that most extant terrestrial animals may have already evolved to be efficient foragers. Given the adaptive losses that these species would experience as a consequence of further enhancements to their locomotion efficiencies^108^, for example longer legs might reduce an animal’s capacity to accelerate^97^, there is little such selective pressure. Harris and Steudel^111^ report that details of prey pursuit and capture are the factors that describe hind limb length, in contrast finding no predictive power in home range size or daily movement distance. Perhaps other physical factors that influence locomotion energy efficiencies, such as leg muscle mass, for similar reasons are also under minimal selection for enhancement in terms of energy efficiencies, particularly in species for which locomotion costs are a relatively small proportion of their total energy expenditure^112^. The possibility that NCOT is simply a poor representation of an animal’s energy costs of locomotion is particularly supported in the present study by the lack of relationship between NCOT and field metabolic rate or activity metabolic rate. NCOT, calculated as the slope of the linear fit between rate of energy expenditure and locomotion speed, tends towards the total energy costs of locomotion per unit distance at high running speeds^33^. However, the majority of movements by animals are conducted at low speeds relative to their maximum obtainable^113–116^. Under these circumstances, per unit distance the cost of transport is higher than indicated by NCOT due to fixed costs associated with locomotion^33^, most notably the ‘postural cost of transport’ PCOT^72^. Furthermore, animals often incur additional energy costs while walking associated with, for example, intermittent locomotion^117^, turning^118^, and negotiating various terrains e.g. Fig. 4B in^119^. NCOT therefore underestimates an animal’s true energy costs to move, and the magnitude of this error presumably varies between species depending upon factors such as their typical running speeds and overall movement behaviours.

To progress our understanding of the energetic costs and constraints for terrestrial animals traversing their habitats, if and how these costs help shape their ecology, and how those costs relate to their locomotion characteristics, perhaps now it is time to step off the laboratory treadmill and measure transport costs in the wild^33,120–122^.

## Electronic supplementary material

Supplementary Dataset 1

## Data Availability

We have made the data collated for the present study available on Dryad.
